# Fast 3D UTE in vivo T_1_ and $T_{2}^{*}$ mapping of fast relaxing knee tissues at 3 T

**DOI:** 10.1002/mrm.70099

**Published:** 2025-10-14

**Authors:** Maik Rothe, Selina Riedel, Anne Slawig, Andreas Deistung, Klaus Bohndorf, Richard Brill, Walter A. Wohlgemuth, Alexander Gussew

**Affiliations:** ^1^ University Clinic and Outpatient Clinic for Radiology University Hospital Halle (Saale) Halle (Saale) Germany; ^2^ Halle MR Imaging Core Facility (HMRICF) Halle (Saale) Germany

**Keywords:** $T_{2}^{*}$ mapping, knee joint, quantitative imaging, T_1_ mapping, UTE

## Abstract

**Purpose:**

UTE MR imaging captures quantitative signals in fast‐relaxing tissues, enabling anatomical visualization and quantitative assessment of T_1_ and $T_{2}^{*}$ relaxation times. However, the clinical application of quantitative UTE MRI is limited by long acquisition times. Therefore, this study introduces a novel UTE‐based method for T_1_ and $T_{2}^{*}$ mapping, achieving submillimeter resolution in less than 10 min.

**Theory and Methods:**

The method employs a dual‐echo acquisition for fast $T_{2}^{*}$ mapping, augmented by an additional acquisition with different T_1_ weighting. This second scan enables the computation of signal ratios between scans with different T_1_‐weighting. These measured signal ratios are then compared to a lookup table containing distinct ratios, corresponding to discrete T_1_ values. The approach was validated in phantom solutions mimicking various T_1_ and $T_{2}^{*}$ times and applied in vivo to quantify relaxation times across different knee tissue compartments in healthy individuals.

**Results:**

The method demonstrated its reliability for T_1_ and $T_{2}^{*}$ quantification in rapidly relaxing tissues (1–11 ms). However, it exhibited a tendency to underestimate $T_{2}^{*}$ in skeletal muscle. This limitation arises from the chosen TEs being inadequate to capture slow signal decays. In accordance with the findings of preceding studies, this in vivo study identified three distinct T_1_ categories of tissue characterized by short (adipose tissue), moderate (ligaments, tendons, and menisci), and long (skeletal muscle) T_1_ values.

**Conclusion:**

The presented technique for combined T_1_ and $T_{2}^{*}$ mapping enables relaxometry in rapidly relaxing tissues, indicating potential for advanced tissue characterization in clinical settings.

## 1 INTRODUCTION

MRI of tendons and ligaments is limited by the rapid transverse relaxation times. Conventional sequences with TEs of several milliseconds yield only weak signals from these tissues. UTE techniques enable imaging of fast‐relaxing tissues.^1^, ^2^, ^3^ Quantitative UTE MRI provides access to proton density, relaxation, and magnetization transfer properties, offering insights into bound and free water fractions in collagen^4^, ^5^, ^6^ and enabling detection of physiological and pathological changes due to aging, strain, or injury.^7^, ^8^

The knee joint represents a clinically relevant target for quantitative UTE imaging owing to its complex anatomy and abundance of fast‐relaxing tissues. Pathological changes in tendons,^9^ ligaments,^10^, ^11^, ^12^, ^13^ cartilage,^14^, ^15^ and menisci^16^ can be characterized by T_1_ and effective $T_{2}^{*}$ relaxation. Whereas $T_{2}^{*}$ is sensitive to local magnetic field inhomogeneities, T_1_ more directly reflects the free water content.^17^ Therefore, mapping of both enables more comprehensive tissue assessment. The anterior cruciate ligament (ACL), for instance, is particularly prone to degenerative or traumatic injury.^18^, ^19^, ^20^ Early detection of scarring or partial tears may improve treatment outcomes.

Recent UTE studies performed exponential fitting of multiple echoes for $T_{2}^{*}$ mapping.^21^, ^22^, ^23^ T_1_ mapping via inversion recovery (IR) is accurate but time‐consuming and suboptimally suited for short $T_{2}^{*}$ tissues due to ineffective inversion. Faster but less precise alternatives include saturation recovery with variable flip angle (FA)^24^, ^25^ or variable TR.^22^ Clinical adoption of UTE relaxometry is limited by long scan times, especially with 3D radial or spiral encoding. Few studies have simultaneously quantified T_1_ and $T_{2}^{*}$ in fast‐relaxing knee tissues,^9^, ^22^, ^26^ typically achieving 2 mm resolution in about 20 min.

In this study, we propose a time‐efficient UTE‐based method for combined T_1_ and $T_{2}^{*}$ mapping of the knee joint at submillimeter isotropic resolution. The method comprises two UTE scans: a dual‐echo acquisition for $T_{2}^{*}$ mapping and a scan with variable T_1_ weighting to enable lookup table (LUT)–based T_1_ quantification.^27^ Validation included phantom experiments and in vivo measurements in healthy volunteers across different knee joint tissues, with comparison to literature values.

## 2 THEORY

**FIGURE 1:**
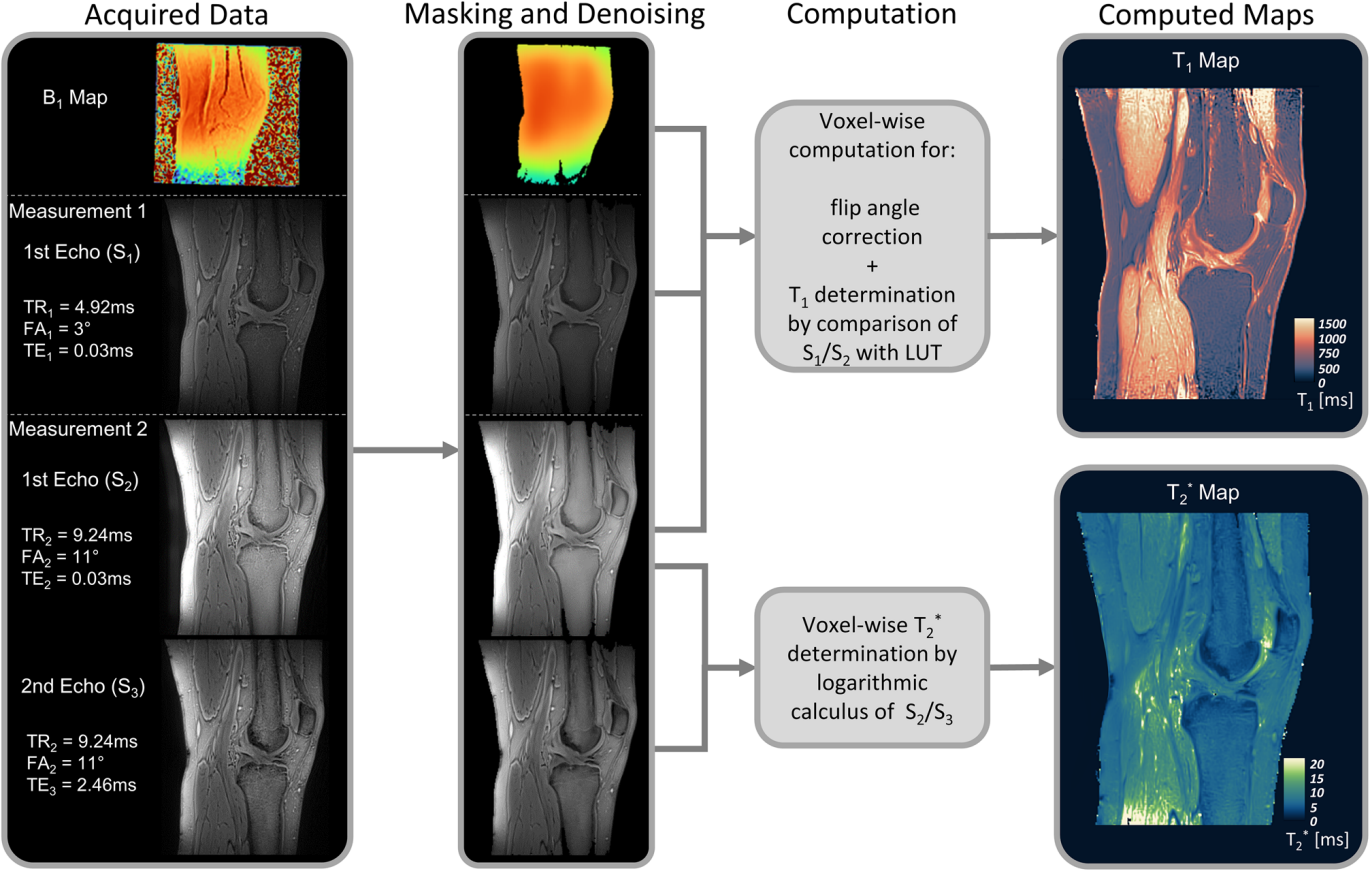
Overview of the proposed method for T_1_ and $T_{2}^{*}$ mapping, illustrated on a sagittal knee image using the Lipari and Navia color maps.^71^ The left column presents the proposed acquisition scheme consisting of two UTE scans and B_1_^+^ mapping. In the subsequent image preprocessing, the background was removed by masking and the SNR was improved by a denoising algorithm (second column). After these steps, the T_1_ and $T_{2}^{*}$ maps were calculated (third column). The right column shows the corresponding $T_{2}^{*}$ map (based on logarithmic calculation of S_2_ and S_3_) as well as the B_1_^+^‐corrected T_1_ map (based on S_1_ and S_2_ via LUT). Peripheral T_1_ inhomogeneity in muscle (e.g., femur) may reflect transmit‐field imperfections at the edge of the FOV. LUT, lookup table; S, signal intensity.

The proposed mapping protocol uses two UTE scans with different parameter settings, complemented by a low‐resolution B_1_^+^ map (Figure 1). The first scan acquires one ultrashort echo (S_1_) with minimized T_1_ contrast. The second scan acquires two echoes (S_2_, S_3_) using parameters optimized for T_1_ sensitivity and for the Ernst‐angle condition in tissues with a T_1_ of approximately 500 ms.

### 2.1 $T_{2}^{*}$ mapping

The spoiled gradient echo signal can be modeled as follows^28^:

(1) $$S=S_{0}\sin\left(\alpha \right)\frac{1-e^{-\frac{T_{R}}{T_{1}}}}{1-e^{-\frac{T_{R}}{T_{1}}}\cos\left(\alpha \right)}e^{-\frac{T_{E}}{T_{2}^{*}}},$$

where S is the signal intensity, $S_{0}$ is the signal intensity at TE = 0 ms, and *α* is the FA.

$T_{2}^{*}$ can be calculated by logarithmic ratio of two echoes acquired in the second scan:

(2) $$T_{2}^{*}=\frac{{\mathrm{TE}}_{3}-{\mathrm{TE}}_{2}}{\ln\left(S_{2}/S_{3}\right)},$$

where S_2_ and S_3_ are signal intensities at ultrashort (TE_2_) and moderate (TE_3_), respectively. TE_3_ is selected to satisfy the in‐phase condition according to the scanner's operating frequency (123.256 MHz at our 3 T system), assuming a chemical shift of 3.3 ppm between water and fat.^29^

Beyond ensuring in‐phase acquisition, TE_3_ must also be matched to the expected $T_{2}^{*}$ range. Long TE_3_ improves accuracy for long $T_{2}^{*}$ values but reduces it for short $T_{2}^{*}$ and vice versa (Figure S1). Details and visualizations of TE_2_/TE_3_ optimization are provided in Figure S1. In this study, $T_{2}^{*}$ mapping was evaluated for two different settings of TE_3_ (2.46 ms and 4.92 ms) and compared to conventional mono‐exponential fitting of three echoes (TEs = 0.03/2.46/4.92 ms).

### 2.2 T_1_ mapping

T_1_ values are derived from two UTE measurements, S_1_ and S_2_, acquired with different FA and/or TR, using the signal model described in Equation 1. Because the ratio S_1_/S_2_ is determined by the underlying T_1_ value, we constructed a LUT that maps discrete S_1_/S_2_ ratios to specific T_1_ values within a predefined range^27^ (1–4000 ms in 1 ms steps in our study):

(3) $$\frac{S_{1}}{S_{2}}=\frac{\sin\left(\alpha_{1}\right)\left(1-e^{-\frac{{\mathrm{TR}}_{1}}{T_{1}}}\right)\left(1-e^{-\frac{{\mathrm{TR}}_{2}}{T_{1}}}\cos\left(\alpha_{2}\right)\right)}{\sin\left(\alpha_{2}\right)\left(1-e^{-\frac{{\mathrm{TR}}_{2}}{T_{1}}}\right)\left(1-e^{-\frac{{\mathrm{TR}}_{1}}{T_{1}}}\cos\left(\alpha_{1}\right)\right)}.$$

Accurate knowledge of the actual FAs is crucial but can be extracted from a low‐resolution B_1_^+^ map.

The choice of FA_1_/FA_2_ and TR_1_/TR_2_ also depends on the expected T_1_ range and is constrained by the settings used for $T_{2}^{*}$ mapping. To reduce scan time, TR_1_ and TR_2_ are kept as short as possible. FA_2_ is selected according to the Ernst angle for tissues with T_1_ ≈ 500 ms. This leaves FA_1_ as the only remaining parameter to be optimized. It must be small enough to minimize T_1_ weighting across a broad T_1_ range, while remaining large enough to ensure adequate SNR in the first UTE scan (S_1_). Based on numerical simulations (Figure S1), FA_1_ = 3° was chosen as a sufficient compromise.

## 3 METHODS

The proposed fast T_1_ and $T_{2}^{*}$ mapping approach was evaluated in vitro and applied in vivo in 20 asymptomatic young volunteers (14/6 female/male, 26.6 ± 6.2 years) with no history of knee trauma, pain, functional impairment, or intense sports activity. The study was approved by the local ethics committee (protocol no.: 2021–056) and conducted in accordance with the Declaration of Helsinki; written informed consent was obtained from all participants.

### 3.1 Phantom construction

Two multi‐compartment phantoms were constructed to mimic T_1_ and $T_{2}^{*}$ relaxation properties in knee tissues. The $T_{2}^{*}$ phantom (seven tubes) was designed to simulate $T_{2}^{*}$ values between 1 ms and 20 ms using 3 wt% carrageenan gels with 0.9 wt% sodium chloride and varying cornstarch concentrations ([80/70/60/50/45/40/33] wt%). Cornstarch effectively shortens $T_{2}^{*}$ without inducing B_0_ field distortions.^30^, ^31^, ^32^ The T_1_ phantom (10 tubes) simulated T_1_ values between 300 and 1300 ms^33^, ^34^ using 3 wt% agarose gels with 0.9 wt% sodium chloride and graded concentrations of gadolinium ([300/180/140/80/60/40/30/20/12/7] μM, gadobutrol).

### 3.2 Data acquisition

MRI was performed on a clinical 3 T MR scanner (Magnetom Vida, Siemens Healthineers, Erlangen, Germany) using an 18‐channel transmit/receive knee coil. In vivo scans were acquired with volunteers in supine position, with knees fixated and angulated by approximately 15°.

The imaging protocol included two UTE scans with 3D stack‐of‐spirals readout (prototype spoiled gradient echo UTE sequence^35^, ^36^) and a low‐resolution B_1_^+^ map (Figure 1). Scans were performed in sagittal orientation with 0.8 mm isotropic resolution (20 μs block‐pulse excitation, FOV: 198 × 198 mm^2^, matrix: 256 × 256, 172 slices, slice thickness: 0.8 mm). Each slice was encoded with 512 spiral readouts (1160 μs readout, 682 samples, spectral bandwidth: 588 kHz, pixel bandwidth: 2298 Hz/pixel, TE range: 30–660 μs from k‐space center to periphery).

- Measurement 1 (3:18 min): TE: 0.03 ms, TR_1_: 4.92 ms, FA_1_: 3°
- Measurement 2 (5:56 min): TE: 0.03/2.46/4.92 ms, TR_2_: 9.24 ms, FA_2_: 11°

B_1_^+^ mapping was performed using a low‐resolution 2D multi‐slice turboFLASH sequence^37^, ^38^ (TR/TE: 29660/2.56 ms, FA_1_/FA_2_: 8/80°, FOV: 200 × 200 mm,^2^ acquisition matrix: 96 × 96, slice thickness: 2 mm). To verify suitability for FA correction, additional experiments in a large cylindrical phantom were performed using the employed B_1_^+^ mapping sequence (Figure S2).

As a reference for $T_{2}^{*}$ quantification across a broad $T_{2}^{*}$ range, the $T_{2}^{*}$ phantom was scanned using an echo‐train shifted multi‐echo (ETsME) approach. Here, 22 measurements were repeated with the first echo shifted from TE_1_: 30–1500 μs, while keeping the subsequent echoes constant (TE_2_‐_5_: 4.92/7.38/9.84/12.3 ms, TR: 13 ms; FA: 12°). Reference T_1_ values of the T_1_ phantom were obtained via inversion‐prepared UTE scans (TE/TR: 0.03/4.4 ms, FA: 6°, 22 TIs: 30–8000 ms) and fitted using three free parameters.

### 3.3 Data preprocessing

The low‐resolution B_1_^+^ map was resampled and aligned to the high‐resolution UTE images using the FreeSurfer package (version 7.4.0) (http://surfer.nmr.mgh.harvard.edu ^39^). Subsequent processing was performed with custom Python scripts using standard libraries.^40^ To mitigate FA uncertainty in fast‐relaxing tissues, the B_1_^+^ map was polynomially smoothed and used to compute voxelwise FA correction factors. UTE images were denoised using an adaptive nonlocal means filtering algorithm^41^ and affinely coregistered. Finally, subtraction images (S_2_–S_3_) were generated to highlight fast‐relaxing tissue structures.

$T_{2}^{*}$ reference values in phantoms were determined via monoexponential fitting of the ETsME decay. $T_{2}^{*}$ mapping in both phantom and in vivo data was performed voxelwise using three different approaches:

- 3TE: Three‐point mono‐exponential fit using TEs: 0.03/2.46/4.92 ms
- 2TE_2.46_: Dual‐echo computation (Equation (2)) using TE_2_/TE_3_: 0.03/2.46 ms
- 2TE_4.92_: Dual‐echo computation (Equation (2)) using TE_2_/TE_3_: 0.03/4.92 ms

T_1_ mapping was performed via a LUT generated from simulated S_1_/S_2_ signal ratios corresponding to T_1_ values between 1 ms and 4000 ms (Figure S1D). To account for B_1_^+^ inhomogeneity, separate LUTs were generated for FAs from 1° to 180° (1° steps). Each voxel's local FA was derived from the B_1_^+^ map and used to select the corresponding LUT. Reference T_1_ values in the phantom were determined by monoexponential fitting of the IR series.

### 3.4 Volumes of interest

Volumes of interest (VOIs) were manually defined using 3D Slicer (https://www.slicer.org ^42^) based on subtraction images (S_2_–S_3_) and transferred to the parameter maps to extract VOI specific mean values of relaxation parameters.

Two circular VOIs per phantom tube were placed on matching slices (~3 pixels from the tube wall) and interpolated across slices to form 3D cylindrical VOIs.

In vivo, VOIs comprising at least 100 voxels were manually segmented for nine knee tissues by consensus of four raters (s.r., m.r., k.b., a.g.) based on sagittal, coronal, and axial views (Figure S3). Tissues included the ACL, posterior cruciate ligament (PCL), patellar tendon (PT), quadriceps tendon (QT), posterior horn of lateral meniscus (LM), bone marrow (BM), infrapatellar fat pad (IFP), subcutaneous adipose tissue (SAT), and skeletal muscle (SM). For the meniscus, tendons, and ligaments, only hyperintense voxels in subtraction images were selected to emphasize fast‐relaxing components; vessels and fascia were excluded whenever possible.

### 3.5 Statistical analysis

Agreement between the proposed and reference methods for $T_{2}^{*}$ and T_1_ quantification in phantoms was evaluated using Bland–Altman analysis. $T_{2}^{*}$ values derived from the 3TE, 2TE_2.46_, and 2TE_4.92_ approaches were assessed in relation to those from ETsME series. LUT‐based T_1_ values were evaluated against values obtained from the IR method. Limits of agreement were defined as the mean difference ± 1.96 SD.

One‐way analysis of variance with Tukey's post hoc test was used to evaluate tissue‐specific differences in in vivo T_1_ and $T_{2}^{*}$ values. $T_{2}^{*}$ assessment was primarily based on 2TE_2.46_ data. A separate analysis of variance tested for method‐related differences in $T_{2}^{*}$ values across tissues.

All analyses were performed using GraphPad Prism (version 10.0.0 for Windows, GraphPad Software, Boston, MA, www.graphpad.com). A *p*‐value <0.05 was considered statistically significant.

## 4 RESULTS

### 4.1 Phantom experiments

**FIGURE 2:**
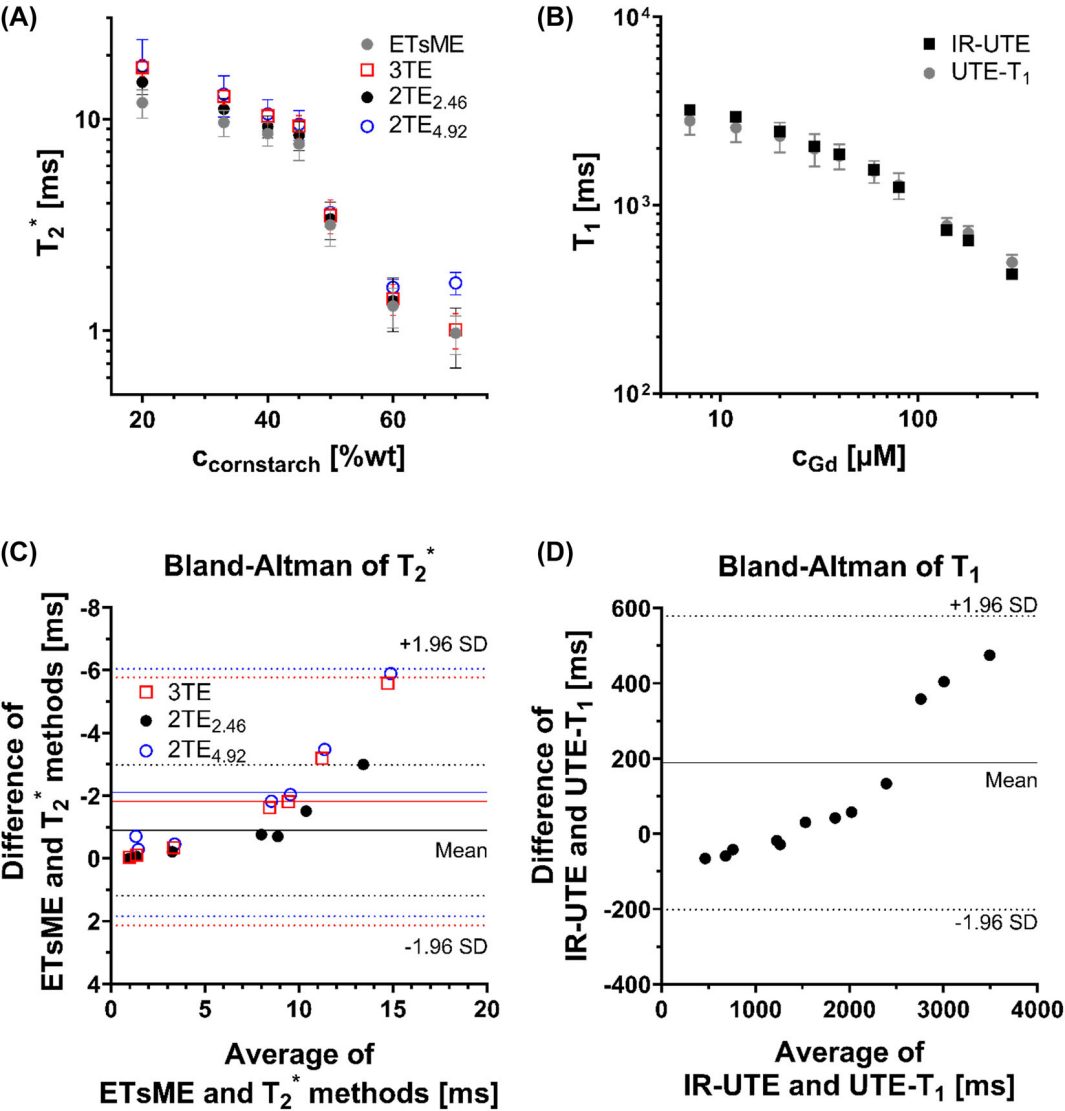
Quantitative mapping in phantom experiments. Mean $T_{2}^{*}$ (A) and T_1_ (B) values are plotted for different concentrations of cornstarch and gadolinium, respectively, for the proposed fast UTE mapping technique and the corresponding reference methods (IR‐UTE for T_1_ and ETsME UTE for $T_{2}^{*}$). The corresponding Bland–Altman plots are shown in the bottom row for $T_{2}^{*}$ (C) and T_1_ (D). The differences in (C) and (D) were calculated as reference method –mapping method. The solid and dotted lines indicate the mean and ± 1.96 SD of the differences between the reference method and the UTE‐based method, respectively (red: 3TE, black: 2TE_2.46_, blue: 2TE_4.92_). ETsME, echo‐train shifted multi‐echo.

As expected, T_1_ and $T_{2}^{*}$ values decreased in the phantoms with increasing gadolinium and cornstarch concentrations, respectively (Figure 2).

$T_{2}^{*}$ values derived from all three UTE‐based methods closely matched ETsME references, showing mean ± SD differences of 16% ± 10% (3TE), 9% ± 7% (2TE_2.46_), and 24% ± 11% (2TE_4.92_) (Figure 2A). Bland–Altman analysis (Figure 2C) demonstrated good agreement up to $T_{2}^{*}$ = 11 ms; beyond this, 2TE_4.92_, and 3 TE increasingly overestimated $T_{2}^{*}$. Additionally, 2TE_4.92_ systematically overestimated values below 3 ms. No reliable values were obtained for $T_{2}^{*}$ < 1 ms.

LUT‐based T_1_ values closely matched IR‐UTE references (Figure 2B), with strong agreement up to T_1_ = 2500 ms (mean difference: 2% ± 7%, maximum deviation: 9%). For T_1_ >2500 ms, LUT estimates exhibited reduced accuracy with a discrepancy of 13% ± 1% (Figure 2D).

### 4.2 In vivo experiments

**FIGURE 3:**
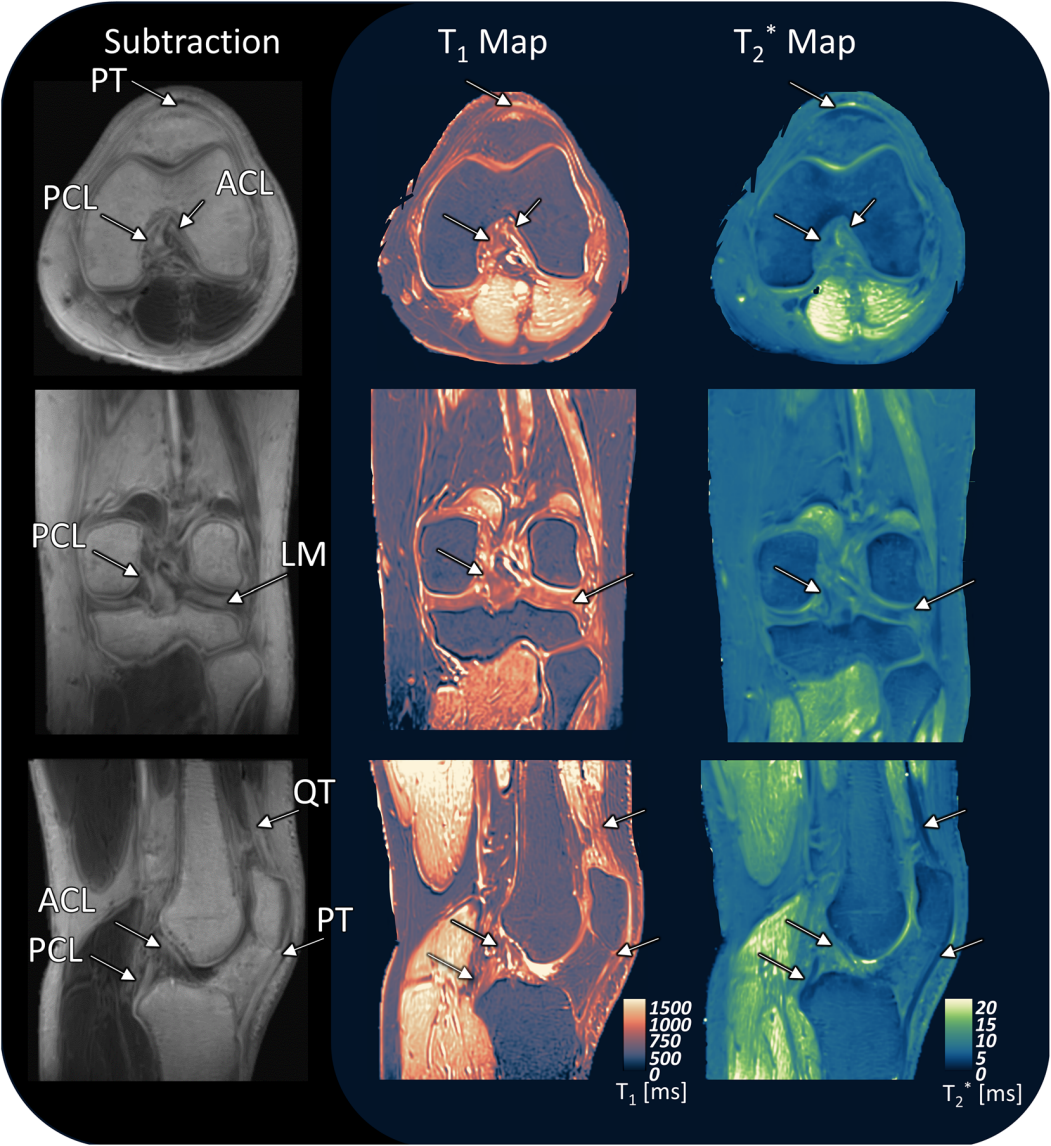
Typical images of the proposed UTE mapping method in three orientations from one volunteer. The subtraction images (S_2_ – S_3_) are shown in the left column, whereas the generated T_1_ maps (displayed in Lipari colormap^71^) and $T_{2}^{*}$ maps from the dual‐echo method (2TE_2.46_, displayed in Navia colormap^71^) are shown in the middle and right columns, respectively. Arrows mark the posterior horn of the LM, ACL, PCL, QT, and PT. ACL, anterior cruciate ligament; LM, lateral meniscus; PCL, posterior cruciate ligament; PT, patellar tendon; S, signal intensity; QT, quadriceps tendon.

**FIGURE 4:**
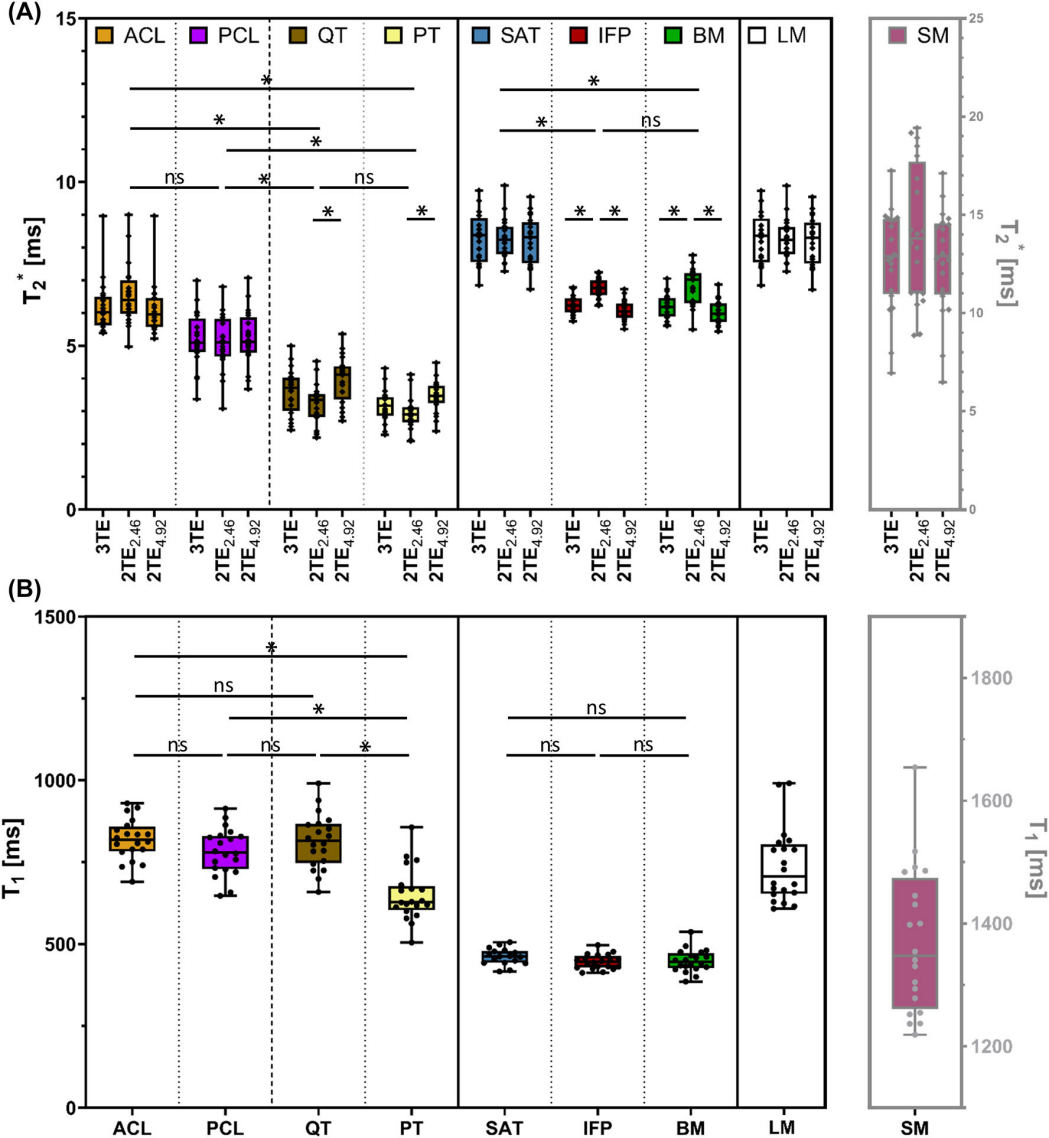
Boxplots of (A) $T_{2}^{*}$ values from three different mapping methods and (B) T_1_ values across nine knee tissue types in 20 healthy volunteers. Each circle represents an individual measurement. Statistical significance between tissue types was assessed using one‐way ANOVA with Tukey's post hoc test (*p < 0.05). ANOVA, analysis of variance; BM, bone marrow; IFP, infrapatellar fat pad; *ns*, not significant; SAT, subcutaneous adipose tissue; SM, skeletal muscle.

Representative UTE subtraction images and corresponding T_1_ and $T_{2}^{*}$ maps are shown in Figure 3, illustrating signal behavior and regional contrast in a healthy knee. Figure 4 and Table 1 summarize the quantitative T_1_ and $T_{2}^{*}$ values across nine examined tissue compartments, alongside literature values.

**TABLE 1:** T_1_ and $T_{2}^{*}$ values for different tissues of the 20 knee joints of healthy volunteers obtained using the proposed imaging protocol compared to literature values.

	T_1_ [s]	T_1_ literature [s]	$T_{2}^{*}$ [ms]			
Tissue			3TE	2TE_2.46_	2TE_4.92_	$T_{2}^{*}$ literature [ms]
Skeletal muscle	1370 ± 120	1060–1420^33^, ^49^, ^61^, ^68^	12.8 ± 2.6	14.0 ± 3.5	12.7 ± 2.7	24–32^56^
Bone marrow	450 ± 35	340–380^24^, ^33^	6.2 ± 0.4	6.8 ± 0.6	6.0 ± 0.4	2–10.3^22^, ^51^, ^52^, ^57^
Infrapatellar fat pad	450 ± 25	370–400^33^, ^68^, ^a^	6.3 ± 0.3	6.8 ± 0.3	6.1 ± 0.4	5–12.5^22^, ^53^, ^54^, ^a^
Subcutaneous adipose tissue	460 ± 25	370–400^33^, ^68^	8.3 ± 0.8	8.3 ± 0.6	8.2 ± 0.7	5–12.5^22^, ^53^, ^54^
Patella tendon	650 ± 85	505–660^22^, ^49^, ^55^, ^61^	3.2 ± 0.5	3.0 ± 0.5	3.5 ± 0.5	1.6–6.4^22^, ^58^, ^60^, ^62^
Quadriceps tendon	810 ± 85	700–800^22^, ^49^	3.6 ± 0.7	3.2 ± 0.6	4.0 ± 0.7	1.4^22^
Posterior cruciate ligament	780 ± 75	710–840^49^, ^55^, ^61^	5.2 ± 0.8	5.1 ± 0.8	5.2 ± 0.8	8.3–8.8^21^, ^55^, ^60^
Anterior cruciate ligament	820 ± 65	740–925^49^, ^55^, ^61^	6.0 ± 0.5	6.3 ± 0.6	6.0 ± 0.5	9.1–16.3^11^, ^55^, ^60^
Lateral Meniscus	740 ± 115	600–970^49^, ^55^, ^61^, ^70^, ^73^	8.3 ± 0.8	8.3 ± 0.7	8.2 ± 0.8	5–10^3^, ^8^, ^16^, ^60^, ^73^

^a^ Assuming same values for infrapatellar fat pad and subcutaneous adipose tissue.

Tendons showed lower $T_{2}^{*}$ values (3–4 ms) than ligaments (5–6 ms), with no significant differences between PT and QT or between ACL and PCL. LM $T_{2}^{*}$ was about 8 ms. Adipose tissues—including SAT, BM, and IFP—had $T_{2}^{*}$ values of 6–8 ms, with significantly higher values in SAT. Interindividual variability (coefficient of variation, CV) was lower in LM and adipose tissues (CV <9%) than in tendons and ligaments (CV: 10%–25%). SM $T_{2}^{*}$ was about half of the literature values (Table 1), with considerable interindividual variability (CV: 10%–25%).

As demonstrated in the $T_{2}^{*}$ phantom experiments, all three mapping methods yielded consistent in vivo values (Figure 4A). However, the 2TE_2.46_ method produced slightly elevated $T_{2}^{*}$ values in the IFP and BM, and lower values in PT and QT.

All measured in vivo T_1_ values were below 2500 ms (Figure 4B), which is within the range validated in the T_1_ phantom. Tissues clustered into three characteristic T_1_ groups: (i) adipose tissue with short T_1_ (400 ms–500 ms, CV: 5%–8%); (ii) tendons, ligaments, and menisci with moderate T_1_ (600 ms–900 ms, CV: 8%–16%); and (iii) SM with a long T_1_ (>1200 ms, CV: 9%). Within the second group, PT showed significantly lower T_1_ than the ligaments (*p* < 0.0001).

## 5 DISCUSSION

We present a framework for fast quantitative UTE MR imaging that enables combined T_1_ and $T_{2}^{*}$ mapping in fast‐relaxing musculoskeletal tissues. All required data were acquired in under 10 min at an isotropic resolution of 0.8 mm^3^. The approach can be further accelerated using techniques such as k‐space undersampling^43^, ^44^ or artificial intelligence‐based superresolution,^45^, ^46^ thereby further enhancing its clinical applicability. This combination of multiparametric mapping, high resolution, and short scan time distinguishes the method from previous approaches.

Typical $T_{2}^{*}$ mapping requires ≥3 echoes and scan times of 9–20 min,^8^, ^21^, ^22^, ^44^, ^47^ often at relatively low (2 mm) or anisotropic resolution, limiting accuracy in small, angled structures such as tendons or menisci due to partial volume effects. Conventional T_1_ mapping using multiple TIs,^48^ variable FAs,^22^, ^49^ or TRs^22^ is similarly time‐consuming (5–20 min) and spatially limited (~2 mm).

Despite these constraints, multi‐parametric approaches—for example, multi‐exponential $T_{2}^{*}$ fits of multiple echoes—can enable more precise differentiation of tissues with distinct relaxation properties but are often impractical for routine clinical use. In contrast, our method specifically targets relevant relaxation ranges via optimized acquisition parameter combinations. The simulations and phantom experiments in this study were designed to define the sensitivity ranges and validate accuracy across different acquisition settings, such as varying TE_3_ in dual‐echo $T_{2}^{*}$ mapping (2TE_2.46_ vs. 2TE_4.92_) or adjusting FA_1_ in the supplemental UTE scan for T_1_ estimation.

LUT‐based T_1_ mapping produced accurate values up to 2500 ms, closely matching the IR‐UTE reference. This confirms that simplified modeling, when paired with tailored acquisition parameters optimized for each relaxation regime, yields robust T_1_ estimates in fast‐ as well as moderate‐to‐long relaxing musculoskeletal tissues.

Phantom experiments demonstrated that selecting 2.46 ms as the moderate TE in the dual‐echo approach enables more accurate $T_{2}^{*}$ values in fast‐relaxing tissues (≤11 ms), with close agreement to reference ETsME fits. Using 4.92 ms led to systematic overestimation for very short $T_{2}^{*}$ values (<3 ms), likely due to excessive signal decay exceeding the measurable dynamic range. Neither method was suitable for extremely short $T_{2}^{*}$ (e.g., cortical bone <1 ms^50^) because signal decay was almost complete at TE = 2.46 ms.

### 5.1 In vivo experiments

As in the phantom experiments, we evaluated 2TE_2.46_, 2TE_4.92_, and 3 TE $T_{2}^{*}$ mapping in knee tissues. For meniscus and adipose tissues, $T_{2}^{*}$ values aligned well with literature references.^3^, ^8^, ^16^, ^21^, ^22^, ^51^, ^52^, ^53^, ^54^, ^55^, ^56^, ^57^ The choice of the moderate echo significantly affected $T_{2}^{*}$ in tendons (*p* < 0.005): 2TE_2.46_ yielded values closest to literature, whereas 2TE_4.92_ and 3 TE increasingly overestimated $T_{2}^{*}$, likely due to advanced signal decay at TE_3_ = 4.92 ms. Even 2TE_2.46_ overestimated $T_{2}^{*}$ in the patellar tendon compared to literature values below 3 ms.^22^, ^58^ This likely reflects the mono‐exponential model's inability to resolve coexisting relaxation components—specifically collagen‐bound and free water—thereby masking the fast‐relaxing component of the signal in collagen‐rich tissue.^59^ Whereas multi‐exponential models can separate such compartments,^58^ they require longer scan times, limiting their clinical practicality.

Other studies reported higher $T_{2}^{*}$ values for ACL and PCL than observed in our cohort,^11^, ^21^, ^55^, ^60^ likely due to different segmentation strategies: We specifically targeted fast‐relaxing compartments that showed high signal in UTE subtraction images (Figure 2), whereas others analyzed entire ligament volumes, including both collagen‐rich and slower‐relaxing regions.^49^, ^55^, ^60^, ^61^

Consistent with previous studies,^11^, ^21^, ^22^, ^55^, ^60^, ^62^ our analysis confirmed lower $T_{2}^{*}$ values in patellar and quadriceps tendons compared to the ACL and PCL, likely due to their higher collagen content (>95% vs. < 90%).^59^ Higher $T_{2}^{*}$ in ligaments may also reflect their greater angulation relative to B_0_, consistent with the “magic angle” effect.^63^, ^64^, ^65^, ^66^, ^67^ In our cohort, tendon and ligament angles ranged between 20° and 35°, which—based on data from Wu et al.^66^ on the Achilles tendon—can increase $T_{2}^{*}$ by several milliseconds.

All applied $T_{2}^{*}$ mapping methods underestimated skeletal muscle values due to limited sensitivity to slow‐relaxing tissues,^56^ necessitating longer TEs for accurate assessment.^8^, ^21^, ^47^

$T_{2}^{*}$ values are anatomy‐ and condition‐dependendent and poorly differentiate collagen‐rich from fatty tissues (Figure 4). In contrast, T_1_—driven by molecular mobility—better reflects tissue properties such as water and macromolecular content. Consistent with previous studies, our results delineate three tissue‐specific T_1_ categories (Figure 4): short T_1_ in adipose tissue (high lipid content)^24^, ^33^, ^68^; moderate T_1_ in collagen‐rich ligaments, tendons, and menisci^49^, ^55^, ^61^, ^69^, ^70^; and prolonged T_1_ in skeletal muscle (elevated free water content).^33^, ^49^, ^61^, ^68^ Notably, patellar and quadriceps tendons also differed in T_1_, consistent with Krämer et al.,^22^ presumably due to differences in free water and collagen content.

Adipose tissues showed higher T_1_ values than literature references (Table 1), likely due to weaker T_1_ weighting in S_1_ for compounds with T_1_ <500 ms (Figure S1C), consistent with phantom data (Figure 2B/D). Lowering FA_1_ in the S_1_ scan could enhance accuracy but reduce SNR, requiring systematic assessment of this tradeoff.

### 5.2 Limitations

A limitation of our T_1_ mapping approach is the vendor‐specific 2D B_1_^+^ map,^38^ which introduces FA uncertainty in fast‐relaxing tissues. Polynomial smoothing was applied to reduce these effects, though small residual errors still may persist. Heterogeneous T_1_ distributions in the FOV periphery (e.g., in bone marrow or muscles; Figure 3) likely reflect incomplete FA correction. This inaccuracy was also evident in phantom measurements, where LUT‐based T_1_ estimates deviated by ~10% from reference IR values near the FOV edge (Figure S2). Therefore, future work should explore B_1_^+^ mapping approaches more closely aligned with UTE protocols.^24^, ^49^

Chemical shift artifacts (~1 pixel) at water–fat boundaries—particularly at muscle–fat interfaces (Figure 1) and in thin structures such as tendons, ligaments, and cortical bone—can affect T_1_ and $T_{2}^{*}$ mapping by introducing heterogeneous signal contributions in boundary voxels due to partial volume and off‐resonance effects. Slightly elevated $T_{2}^{*}$ values in tissues such as the patellar tendon may partly reflect lipid contamination. Fat suppression (e.g., Dixon, spectral saturation) could mitigate this but was not applied due to: (1) reduced efficiency from B_0_ inhomogeneities, (2) prolonged scan time, and (3) limited spectral separation at short readouts (~1.16 ms). Future work should therefore develop and evaluate fat suppression techniques optimized for UTE relaxometry.

In stack‐of‐spirals UTE, the effective TE varies across k‐space, from 30 μs centrally to >600 μs peripherally, due to the increasing duration of slice‐encoding gradients. Whereas most signal is acquired at short TE, this spread can bias $T_{2}^{*}$ estimates for ultrashort components (<2 ms), thereby reducing contrast or blurring fast‐decaying signals. Thus, radial 3D UTE trajectories, which maintain uniform TE across k‐space, may offer a more robust alternative for future studies.

## 6 CONCLUSION

We present a fast framework for submillimeter T_1_ and $T_{2}^{*}$ mapping of fast‐relaxing tissues. Whole‐knee coverage was achieved in under 10 min, providing accurate estimates for T_1_ up to 2500 ms and $T_{2}^{*}$ from 1 ms to 11 ms. T_1_ mapping effectively differentiated adipose, collagen‐rich, and water‐rich tissues. Combined T_1_/$T_{2}^{*}$ mapping improves overall tissue characterization and may enhance the assessment of small structures such as ligaments and tendons in future studies.

## Supporting information


**Figure S1.** Simulation‐based optimization of echo times and flip angles for dual‐echo $T_{2}^{*}$ and LUT‐based T_1_ mapping in UTE MRI. (A) Simulated gradient‐echo signal decay curves for different $T_{2}^{*}$ values. Vertical lines mark the ultrashort (0.03 ms) and the first two in‐phase echoes at 3 T (2.46 ms, 4.92 ms), assuming a 3.3 ppm water–fat chemical shift at 123.256 MHz. In this study, TE_2_ = 0.03 ms and TE_3_ = 2.46 ms or 4.92 ms were used. (B) Ratio S_2_/S_3_ as a function of $T_{2}^{*}$ for different TE_3_ values (TE_2_ fixed at 0.03 ms). The shaded area indicates the optimal sensitivity range: S_3_ decayed by ≥25% for better discrimination of longer $T_{2}^{*}$, but retained >5% of S_2_ to reduce noise sensitivity. (C) Simulated gradient‐echo signals for ultrashort TE = 0.03 ms with varying T_1_ and FA. S_2_ (TR = 9.24 ms, FA_2_ = 11°) corresponds to the $T_{2}^{*}$ mapping scan; S_1_ (TR = 4.92 ms, FA_1_ = 1–6°) corresponds to the first UTE scan with minimized T_1_ contrast. (D) Ratio S_1_/S_2_ versus T_1_ for different FA_1_ values. The dashed line marks FA_1_ = 3°, chosen as a compromise between minimal T_1_ weighting across a wide T_1_ range and adequate SNR in S_1_. Shaded limits indicate where S_1_'s T_1_ weighting falls below the noise level of S_2_ or where S_1_ and S_2_ are equal within noise.

**Figure S2.** Effects of B_1_^+^‐correction on T_1_‐mapping. Top left: Direct comparison of T_1_‐mapping without B_1_^+^‐correction, with B_1_^+^‐correction using the dual‐angle (DA) approach (as used in this study), with B_1_^+^‐correction using the Actual Flip angle Imaging (AFI) B_1_^+^‐mapping approach, and with a gold‐standard IR‐UTE‐based T_1_‐mapping experiment. Top right: Three‐plane view of the used phantom with a known T_1_ of 100 ms^72^ (top: axial view; middle: coronal view; bottom: sagittal view). The cylindrical phantom has a height of 20 cm and a diameter of 13 cm and consists of: 3.75 g *NiSO*_*4*_ and 5 g NaCl per 1000 g H_2_O. Six different cubic volumes (15 mm × 15 mm × 15 mm) were positioned in the isocenter along the direction of the main magnetic field. Bottom first row: B_1_^+^ maps obtained with the AFI (left) and DA (right) methods, displayed in % of nominal FA. The yellow box marks the cropped phantom region used for all maps and includes an overlay of a representative in vivo knee image (50% transparency) to illustrate correspondence with the in vivo field of view. Bottom second row: Corresponding T_1_ maps are shown without B_1_^+^ correction, with DA correction, and with AFI correction. Uncorrected maps exhibit pronounced spatial inhomogeneity, which is reduced by either correction approach.

**Figure S3.** Visualization of manually segmented regions for the patellar tendon, quadriceps tendon, anterior cruciate ligament (ACL), posterior cruciate ligament (PCL), infrapatellar fat pad, subcutaneous adipose tissue, bone marrow, skeletal muscle and the posterior horn of the lateral meniscus. Segments are overlaid on UTE subtraction images (S_1_ – S_2_) of a knee in axial and three sagittal views. All three planes (axial, coronal, and sagittal) were used for tissue segmentation, as exemplified for the ACL in the three images in the center column of the bottom row. Numbers indicate the planes specified by the dashed lines in the upper left corner.
