# Supplementary material for: Fast 3D UTE in vivo T1 and T2* mapping of fast relaxing knee tissues at 3 T
Source: Magn Reson Med. 2025 Oct 14;95(2):693–705. doi: 10.1002/mrm.70099 (PMC12681309; doi:10.1002/mrm.70099)
Supplement: Supplementary file 1 — Figure S1. Simulation‐based optimization of echo times and flip angles for dual‐echo T2* and LUT‐based T1 mapping in UTE MRI. (A) Simulated gradient‐echo signal decay curves for different T2* values. Vertical lines mark the ultrashort (0.03 ms) and the first two in‐phase echoes at 3 T (2.46 ms, 4.92 ms), assuming a 3.3 ppm water–fat chemical shift at 123.256 MHz. In this study, TE2 = 0.03 ms and TE3 = 2.46 ms or 4.92 ms were used. (B) Ratio S2/S3 as a function of T2* for different TE3 values (TE2 fixed at 0.03 ms). The shaded area indicates the optimal sensitivity range: S3 decayed by ≥25% for better discrimination of longer T2*, but retained >5% of S2 to reduce noise sensitivity. (C) Simulated gradient‐echo signals for ultrashort TE = 0.03 ms with varying T1 and FA. S2 (TR = 9.24 ms, FA2 = 11°) corresponds to the T2* mapping scan; S1 (TR = 4.92 ms, FA1 = 1–6°) corresponds to the first UTE scan with minimized T1 contrast. (D) Ratio S1/S2 versus T1 for different FA1 values. The dashed line marks FA1 = 3°, chosen as a compromise between minimal T1 weighting across a wide T1 range and adequate SNR in S1. Shaded limits indicate where S1's T1 weighting falls below the noise level of S2 or where S1 and S2 are equal within noise. [file MRM-95-693-s002.docx]

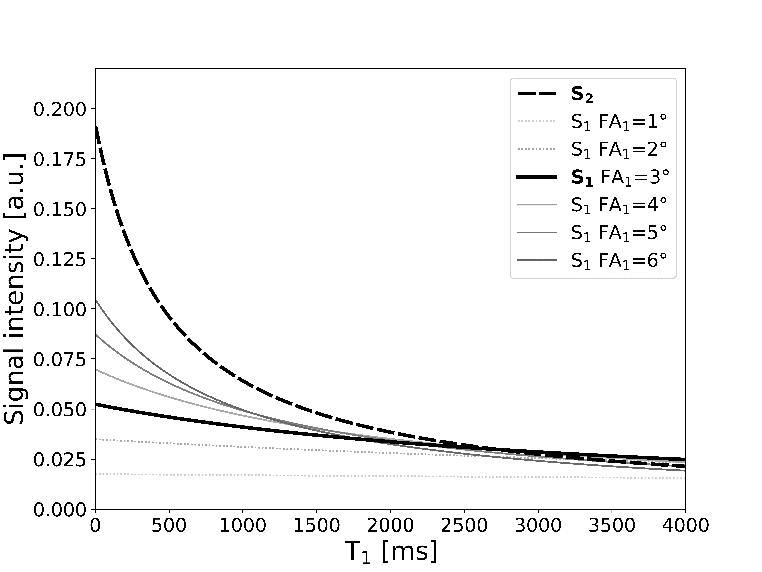

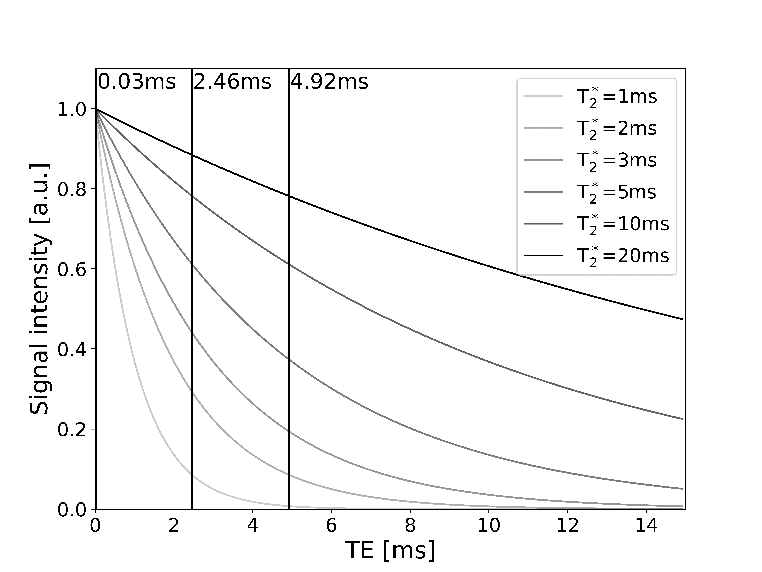

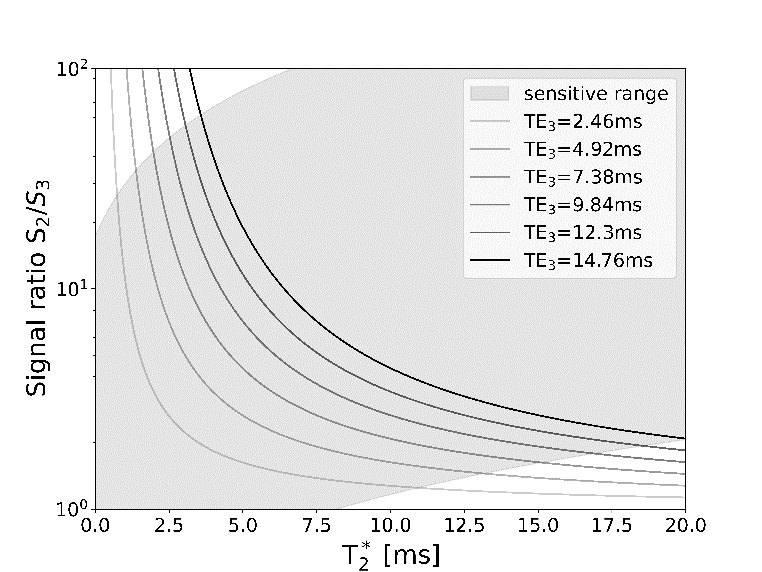

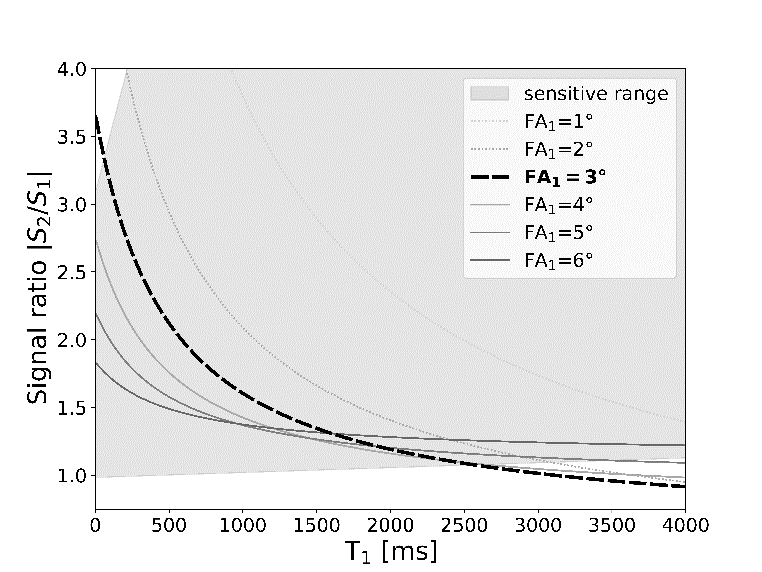


Supplemental Figure 1: Simulation-based optimization of echo times and flip angles for dual-echo T_2_^*^ and LUT-based T_1_ mapping in UTE MRI. (A) Simulated gradient‐echo signal decay curves for different T_2_^*^ values. Vertical lines mark the ultrashort (0.03 ms) and the first two in‐phase echoes at 3 T (2.46 ms, 4.92 ms), assuming a 3.3 ppm water–fat chemical shift at 123.256 MHz. In this study, TE_2_ = 0.03 ms and TE_3_ = 2.46 ms or 4.92 ms were used. (B) Ratio S_2_/S_3_ as a function of T_2_^*^ for different TE_3_ values (TE_2_ fixed at 0.03 ms). The shaded area indicates the optimal sensitivity range: S_3_ decayed by ≥25% for better discrimination of longer T_2_^*^, but retained >5% of S_2_ to reduce noise sensitivity. (C) Simulated gradient‐echo signals for ultrashort TE = 0.03 ms with varying T_1_ and FA. S_2_ (TR = 9.24 ms, FA_2_ = 11°) corresponds to the T_2_^*^ mapping scan; S_1_ (TR = 4.92 ms, FA_1_ = 1–6°) corresponds to the first UTE scan with minimized T_1_ contrast. (D) Ratio S_1_/S_2_ versus T_1_ for different FA_1_ values. The dashed line marks FA_1_ = 3°, chosen as a compromise between minimal T_1_ weighting across a wide T_1_ range and adequate SNR in S_1_. Shaded limits indicate where S_1_’s T_1_ weighting falls below the noise level of S_2_ or where S_1_ and S_2_ are equal within noise.
