# Supplementary material for: Fast 3D UTE in vivo T1 and T2* mapping of fast relaxing knee tissues at 3 T
Source: Magn Reson Med. 2025 Oct 14;95(2):693–705. doi: 10.1002/mrm.70099 (PMC12681309; doi:10.1002/mrm.70099)
Supplement: Supplementary file 2 — Figure S2. Effects of B1 +‐correction on T1‐mapping. Top left: Direct comparison of T1‐mapping without B1 +‐correction, with B1 +‐correction using the dual‐angle (DA) approach (as used in this study), with B1 +‐correction using the Actual Flip angle Imaging (AFI) B1 +‐mapping approach, and with a gold‐standard IR‐UTE‐based T1‐mapping experiment. Top right: Three‐plane view of the used phantom with a known T1 of 100 ms 72 (top: axial view; middle: coronal view; bottom: sagittal view). The cylindrical phantom has a height of 20 cm and a diameter of 13 cm and consists of: 3.75 g NiSO 4 and 5 g NaCl per 1000 g H2O. Six different cubic volumes (15 mm × 15 mm × 15 mm) were positioned in the isocenter along the direction of the main magnetic field. Bottom first row: B1 + maps obtained with the AFI (left) and DA (right) methods, displayed in % of nominal FA. The yellow box marks the cropped phantom region used for all maps and includes an overlay of a representative in vivo knee image (50% transparency) to illustrate correspondence with the in vivo field of view. Bottom second row: Corresponding T1 maps are shown without B1 + correction, with DA correction, and with AFI correction. Uncorrected maps exhibit pronounced spatial inhomogeneity, which is reduced by either correction approach. [file MRM-95-693-s003.docx]

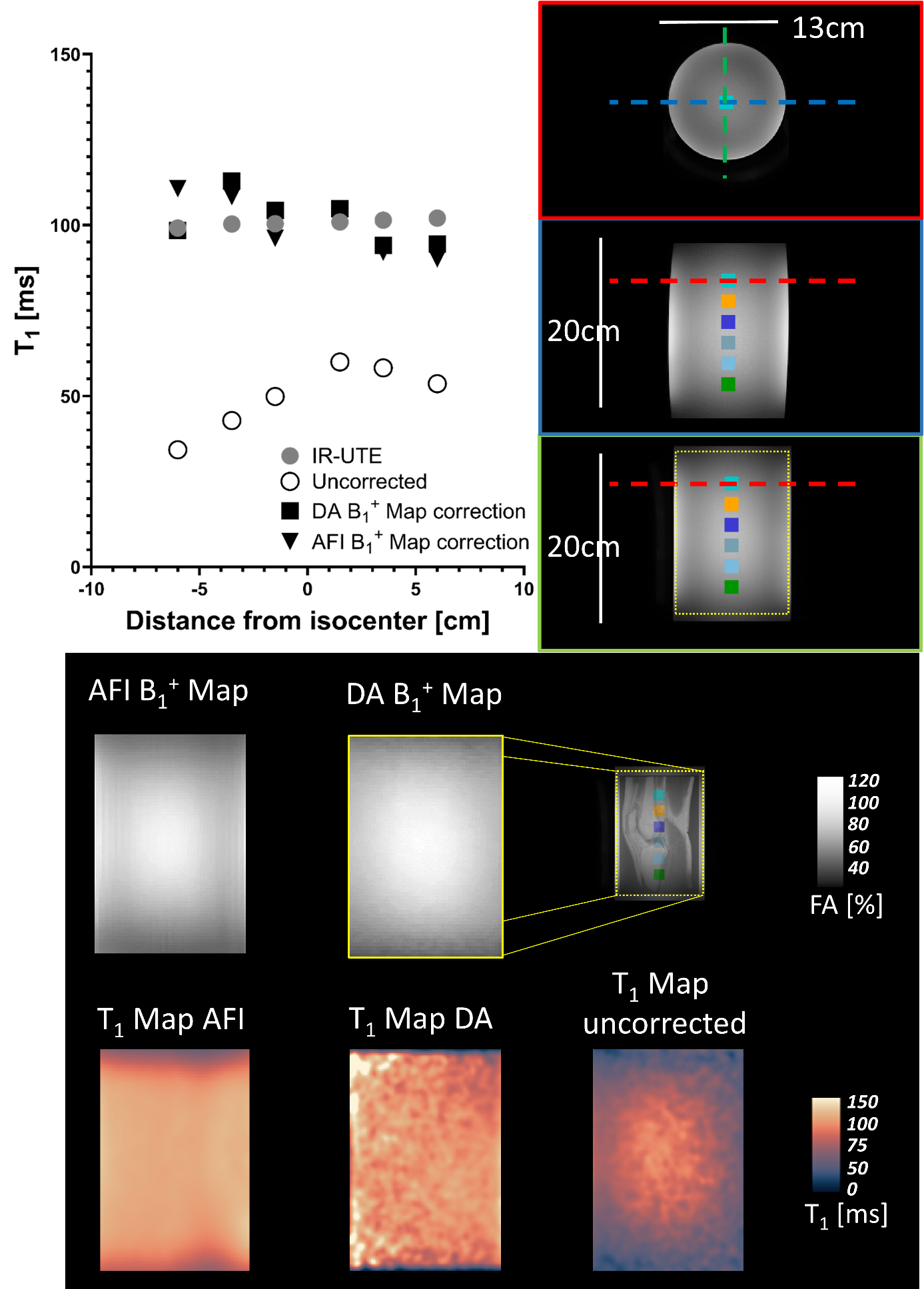


Supplemental Figure 2: Effects of B_1_^+^-correction on T_1_-mapping. Top left: Direct comparison of T_1_-mapping without B_1_^+^-correction, with B_1_^+^-correction using the dual-angle (DA) approach (as used in this study), with B_1_^+^-correction using the Actual Flip angle Imaging (AFI) B_1_^+^-mapping approach, and with a gold-standard IR-UTE-based T_1_-mapping experiment. Top right: Three-plane view of the used phantom with a known T_1_ of 100 ms^72^ (top: axial view; middle: coronal view; bottom: sagittal view). The cylindrical phantom has a height of 20 cm and a diameter of 13 cm and consists of: 3.75 g NiSO_4_ and 5 g NaCl per 1000 g H_2_O. Six different cubic volumes (15 mm × 15 mm × 15 mm) were positioned in the isocenter along the direction of the main magnetic field. Bottom first row: B_1_^+^ maps obtained with the AFI (left) and DA (right) methods, displayed in % of nominal FA. The yellow box marks the cropped phantom region used for all maps and includes an overlay of a representative *in vivo* knee image (50% transparency) to illustrate correspondence with the in vivo field of view. Bottom second row: Corresponding T_1_ maps are shown without B_1_^+^ correction, with DA correction, and with AFI correction. Uncorrected maps exhibit pronounced spatial inhomogeneity, which is reduced by either correction approach.
