# Supplementary material for: Fast 3D UTE in vivo T1 and T2* mapping of fast relaxing knee tissues at 3 T
Source: Magn Reson Med. 2025 Oct 14;95(2):693–705. doi: 10.1002/mrm.70099 (PMC12681309; doi:10.1002/mrm.70099)
Supplement: Supplementary file 3 — Figure S3. Visualization of manually segmented regions for the patellar tendon, quadriceps tendon, anterior cruciate ligament (ACL), posterior cruciate ligament (PCL), infrapatellar fat pad, subcutaneous adipose tissue, bone marrow, skeletal muscle and the posterior horn of the lateral meniscus. Segments are overlaid on UTE subtraction images (S1 – S2) of a knee in axial and three sagittal views. All three planes (axial, coronal, and sagittal) were used for tissue segmentation, as exemplified for the ACL in the three images in the center column of the bottom row. Numbers indicate the planes specified by the dashed lines in the upper left corner. [file MRM-95-693-s001.docx]

**2**

**3**

3

1

2

Lateral Meniscus

Patella tendon

ACL

PCL

Infrapatellar fat pad

Sub. adipose tissue

Bone marrow

Skeletal muscle


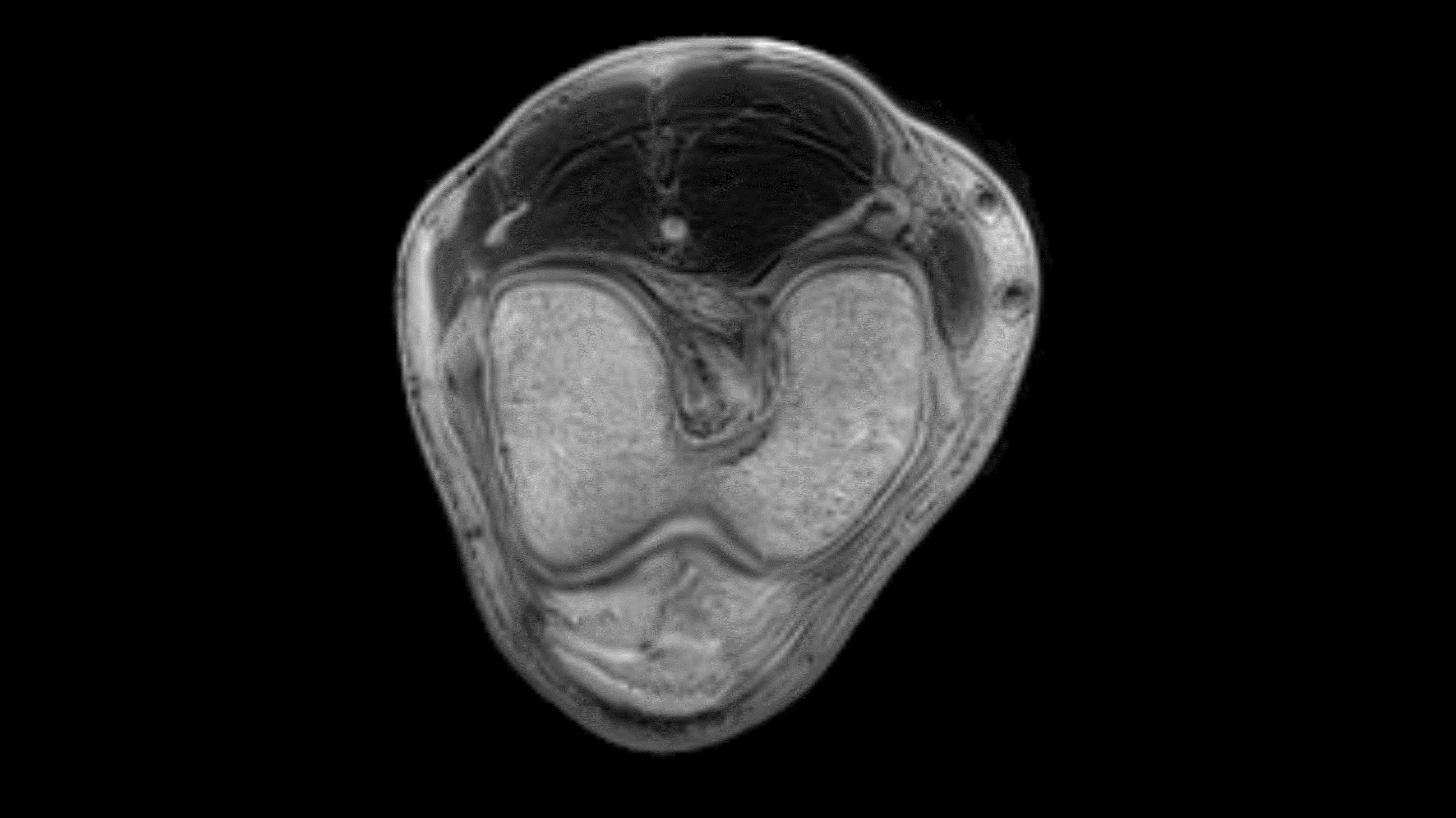


4

**1**


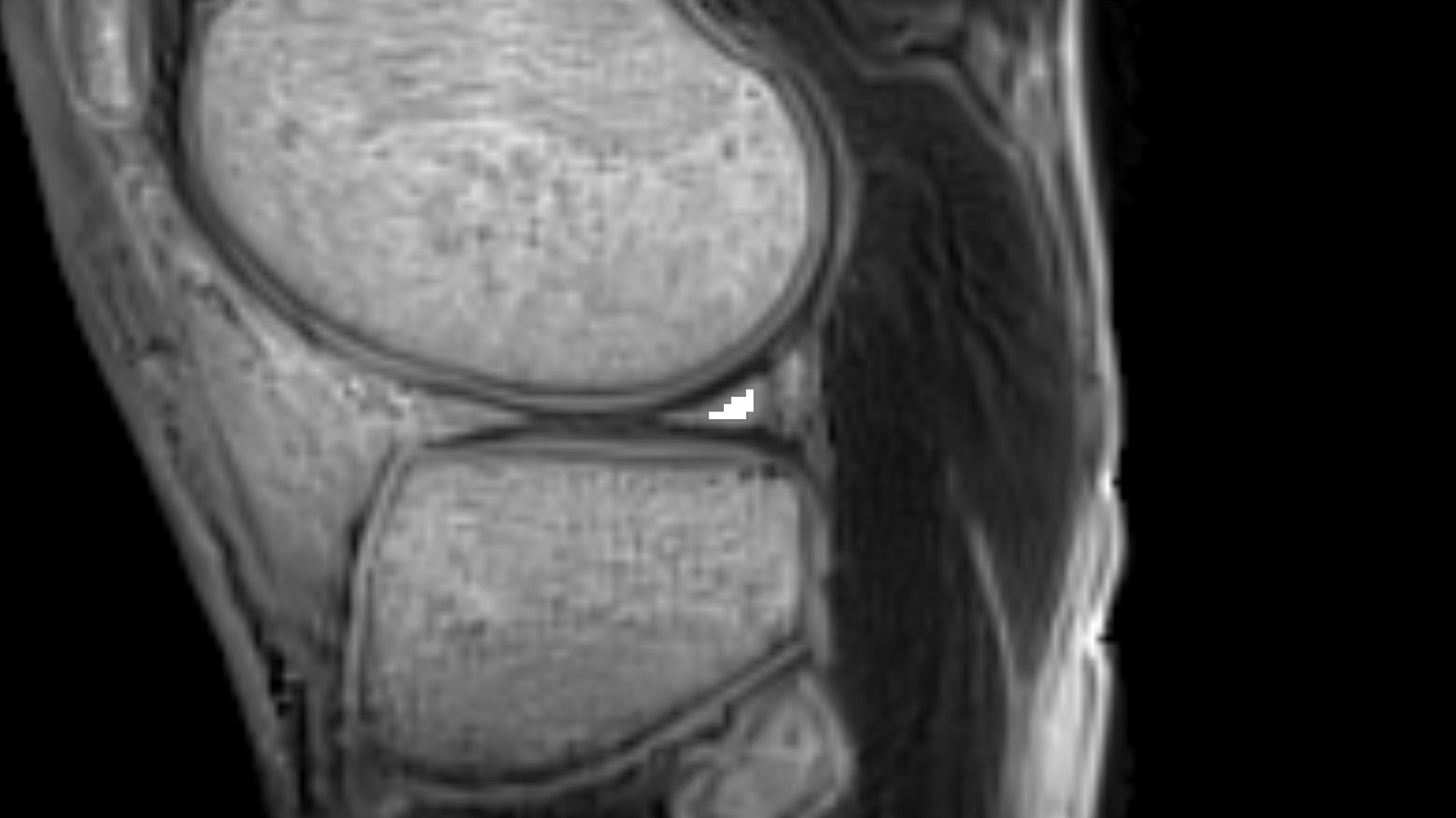


**4**

**3**


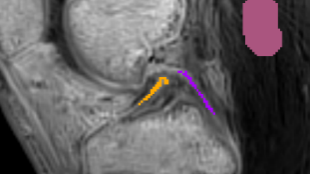

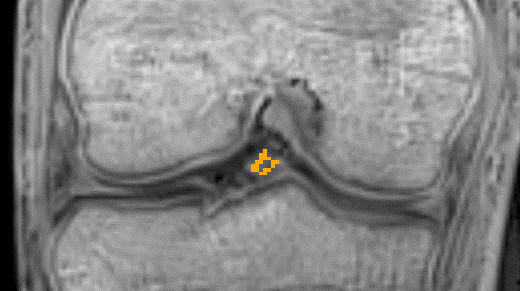

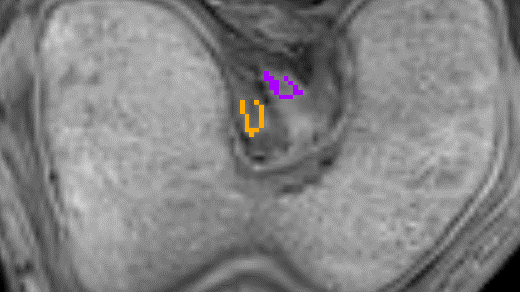

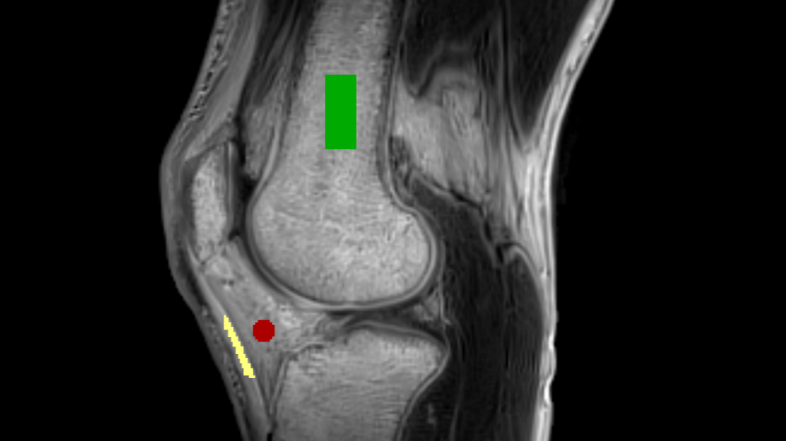


Quadriceps tendon


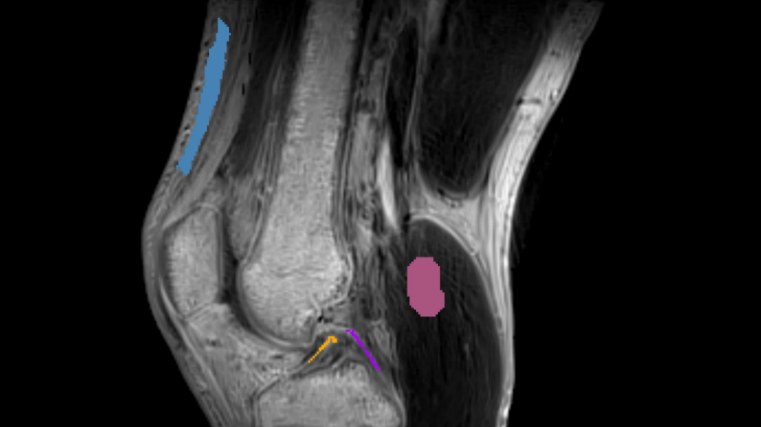


5

**5**

Supplemental Figure 3: Visualization of manually segmented regions for the patellar tendon, quadriceps tendon, anterior cruciate ligament (ACL), posterior cruciate ligament (PCL), infrapatellar fat pad, subcutaneous adipose tissue, bone marrow, skeletal muscle and the posterior horn of the lateral meniscus. Segments are overlaid on UTE subtraction images (S_1_ – S_2_) of a knee in axial and three sagittal views. All three planes (axial, coronal, and sagittal) were used for tissue segmentation, as exemplified for the ACL in the three images in the center column of the bottom row. Numbers indicate the planes specified by the dashed lines in the upper left corner.
